# Adherence to the healthy lifestyle guideline in relation to the metabolic syndrome: Analyses from the 2013 and 2018 Indonesian national health surveys

**DOI:** 10.1016/j.pmedr.2022.101806

**Published:** 2022-04-27

**Authors:** Fathimah S. Sigit, Stella Trompet, Dicky L. Tahapary, Dante S. Harbuwono, Saskia le Cessie, Frits R. Rosendaal, Renée de Mutsert

**Affiliations:** a Department of Clinical Epidemiology, Leiden University Medical Center, Albinusdreef 2, 2333 ZA Leiden, the Netherlands; b Metabolic, Cardiovascular, and Aging Cluster, Indonesia Medical Education and Research Institute, Faculty of Medicine – Universitas Indonesia, Jalan Salemba Raya No 6, Jakarta 10430, Indonesia; c Department of Internal Medicine, Section of Gerontology and Geriatrics, Leiden University Medical Center, Albinusdreef 2, 2333 ZA Leiden, the Netherlands; d Department of Internal Medicine, Dr. Cipto Mangunkusumo National Referral Hospital, Faculty of Medicine – Universitas Indonesia, Jalan Salemba Raya No 6, Jakarta 10430, Indonesia; e Ministry of Health, Republic of Indonesia, Jalan H.R. Rasuna Said Blok X.5 Kav. 4-9, Indonesia

**Keywords:** Metabolic syndrome (MetS), Healthy lifestyle, Adherence, ‘GERMAS’ guideline, Indonesia

## Abstract

In this study, we aimed to investigate differences in lifestyle factors and prevalence of metabolic syndrome (MetS) in the Indonesian population between 2013 and 2018. In addition, we investigated whether adherence to the 2015-released national healthy lifestyle guideline (‘GERMAS’) is associated with MetS in different sex, age, urban/rural, and BMI categories. We performed cross-sectional analyses in individuals aged >15 of the 2013 (n = 34,274) and 2018 (n = 33,786) Indonesian National Health Surveys. A stratified, multi-stage, systematic random sampling design and the probability proportional to size method were used to select households in the 34 provinces across the country*.* MetS was defined according to the Joint Interim Statement Criteria, and adherence to ‘GERMAS’ guideline was defined as fulfilling the national healthy lifestyle recommendations of ≥150 min/week physical activity (PA), ≥5 portions/day fruit and vegetable (FV), no smoking (NS), and no alcohol consumption (NA). We examined the associations of each lifestyle factor with MetS using logistic regression categorised by sex, age groups, urban/rural, and BMI, and adjusted for sociodemographic factors. We observed that men who adhered to the guideline had lower odds ratio of MetS [OR(95%CI) associated with PA: 0.85(0.75–0.97); NA: 0.75(0.56–1.00)] than non-adherent men. Middle-aged adults who adhered to the guideline had lower OR of MetS [PA: 0.85(0.72–1.01); FV: 0.78(0.62–0.99); NA: 0.66(0.46–0.93)] than non-adherent adults <45 years. The adherent urban population had lower OR of MetS [FV: 0.85(0.67–1.07); NA: 0.74(0.52–1.07)] than the non-adherent urban population. Those with overweight or obesity who adhered to the guideline had relatively lower odds of MetS than those who did not. In conclusion, in this nationally representative study, adherence to the ‘GERMAS’ guideline may confer cardiometabolic health benefits to several groups of the Indonesian population, particularly men, middle-aged, those with overweight and obesity, and potentially urban population.

## 1 Introduction

Metabolic syndrome is a strong risk factor for ischemic heart disease, cerebrovascular disease, and diabetes (Alberti et al., 2009, International Diabetes Federation, 2021, Grundy et al., 2005, Tchernof and Després, 2013), which are the three leading causes of disability-adjusted life years (DALYs) in Indonesia (Mboi et al., 2018). In our previous study analysing the 2013 Indonesian National Health Survey, we observed that metabolic syndrome was present in 39% of the middle-aged population (Sigit et al., 2020). In 2015, as an effort to eliminate the nation’s growing burden of both non-communicable and infectious disease, the Indonesian Ministry of Health released a national public health guideline to promote a healthy lifestyle (‘*Gerakan Masyarakat Hidup Sehat; GERMAS’*) (GERMAS, 2021).

The ‘GERMAS’ guideline consists of seven recommendations, which are: (i) regular physical activity, (ii) adequate fruit and vegetable intake, (iii) no smoking, (iv) no alcohol consumption, (v) routine health examination, (vi) preserving environmental cleanliness, and (vii) personal hygiene (GERMAS, 2021). During 2016–2018, extensive nationwide health campaigns were performed to introduce the guideline, particularly focusing on physical activity, fruit and vegetable consumption, and health examination (GERMAS, 2021, Kementerian Kesehatan Republik Indonesia, 2017, Kementerian Kesehatan Republik Indonesia, 2016, Kementerian Kesehatan Republik Indonesia, 2016). The national campaign was supported by the implementation of local/regional policies to actively promote the guideline at the provincial level (Kementerian Kesehatan Republik Indonesia, 2018, Kementerian Kesehatan Republik Indonesia, 2019). It is yet unknown whether the extensive health promotion may have influenced lifestyle behaviour in the population. It is also unclear whether adherence to the guideline may help to prevent the metabolic syndrome in the population.

It is well-established that lifestyle has a substantial impact on general health (Li et al., 2020, Pate et al., 1995, Sofi et al., 2008, Ruitenberg et al., 2002, Inoue-Choi et al., 2017, Will et al., 2001, Lee et al., 2017, Warburton and Bredin, 2017). However, in relation to metabolic syndrome, lifestyle studies from various populations observed different associations. For example, studies in Spanish and North-American populations showed that higher adherence to healthy lifestyle guidelines was associated with lower risks of metabolic syndrome (Garralda-Del-Villar et al., 2018, VanWormer et al., 2017, Hershey et al., 2021, Sotos-Prieto et al., 2021). Conversely, studies in an African-American population found no association between lifestyle behaviour (physical activity, cigarette smoking, and alcohol drinking) and metabolic syndrome (Bhanushali et al., 2013). This discrepancy may partly be due to diverse study designs (cross-sectional/cohort) and diagnostic criteria of metabolic syndrome (ATP-III/Joint Interim Statement), limited adjustment for confounding, and some studies only had relatively few subjects. Large population-based studies are therefore warranted to estimate the presence and strength of the association between adopting a healthy lifestyle with the risk of metabolic syndrome. To date, there are no published studies showing how combined lifestyle factors are associated with the metabolic syndrome in the Indonesian population.

Previous studies have also established that sociodemographic factors were associated with the metabolic syndrome. Studies in various populations have shown that the prevalence of metabolic syndrome differs considerably between men and women (Park et al., 2004, Wang et al., 2020, Gouveia et al., 2021, Yi and An, 2020, Aekplakorn et al., 2011, Dallongeville et al., 2005, Song et al., 2015, Weng et al., 2007), between age categories (Park et al., 2004, Wang et al., 2020, Gouveia et al., 2021, Yi and An, 2020), and between urban or rural living situations (Park et al., 2004, Wang et al., 2020, Song et al., 2015, Weng et al., 2007, Abdul-Rahim et al., 2001, Arambepola et al., 2008, Han et al., 2018, Lindroth et al., 2014). Whether the associations of lifestyle with metabolic syndrome may differ in these groups of individuals in the large and heterogeneous population of Indonesia are unknown.

Therefore, we had two objectives for this study. First, as national health promotion was performed to introduce the ‘GERMAS’ guideline during 2016–2018 (GERMAS, 2021, Kementerian Kesehatan Republik Indonesia, 2017, Kementerian Kesehatan Republik Indonesia, 2016, Kementerian Kesehatan Republik Indonesia, 2016), we aimed to investigate the differences in lifestyle behaviour and prevalence of metabolic syndrome in the Indonesian population between 2013 and 2018. Second, using the 2018 population survey, we aimed to investigate the associations of adherence to the guideline with the metabolic syndrome, and how these associations may differ between sex, age groups, body mass index (BMI), and urban/rural categories.

## 2 Methods

### 2.1 Study design and population

This study consists of cross-sectional analyses of the 2013 and 2018 Indonesian National Health Surveys (Indonesian: *‘Riset Kesehatan Dasar; RISKESDAS’*). RISKESDAS is a routine survey conducted by the Indonesian Government every five years, with the 2013 and 2018 surveys as the two most recent. It was designed to monitor the health status of the citizens, particularly to screen for the presence of infectious, metabolic, and degenerative diseases. A stratified, multi-stage, systematic random sampling design and the probability proportional to size method were used to select households in the 34 provinces across the country. Weighting factors were calculated to ensure that samples were representative of the different geographical densities and urban/rural distribution among the 34 provinces (Kementerian Kesehatan Republik Indonesia, 2013, National Institute for Health Research and Development (NIHRD), Ministry of Health, Republic of Indonesia. Laporan Nasional RISKESDAS, 2013, Kementerian Kesehatan Republik Indonesia, 2018, National Institute for Health Research and Development (NIHRD), Ministry of Health, Republic of Indonesia. Laporan Nasional RISKESDAS, 2018).

Although the 2013 and 2018 surveys had a similar study design, they were two separate cross-sectional surveys. The 2013 survey sampled 1,027,763 participants of all ages (n = 1,105,593 invited; response rate 93.0%), including 722,329 adults aged ≥15 years (National Institute for Health Research and Development (NIHRD), Ministry of Health, Republic of Indonesia. Laporan Nasional RISKESDAS, 2013). The 2018 survey population were 1,017,290 individuals (n = 1,091,528 invited; response rate 93.2%), with 713,783 aged ≥15 years (National Institute for Health Research and Development (NIHRD), Ministry of Health, Republic of Indonesia. Laporan Nasional RISKESDAS, 2018). The present study included non-pregnant adults aged ≥15 years who were randomly sampled for blood lipid and glucose examinations as follows: first, a subsample from the total participants was selected randomly for blood lipid tests and information on lifestyle factors was collected from the participants (n = 34,274 in the 2013 survey; n = 33,786 in the 2018 survey). This was followed by a random selection of a subsample from these participants who then undergo blood glucose test and information on the components of metabolic syndrome was collected (n = 26,160 in the 2013 survey, n = 24,451 in the 2018 survey). A study flow chart illustrating the inclusion criteria of the study is available at **[**Supplemental Fig. 1**].**

The 2013 and 2018 RISKESDAS methodology were described comprehensively in previous government publications (Kementerian Kesehatan Republik Indonesia, 2013, National Institute for Health Research and Development (NIHRD), Ministry of Health, Republic of Indonesia. Laporan Nasional RISKESDAS, 2013, Kementerian Kesehatan Republik Indonesia, 2018, National Institute for Health Research and Development (NIHRD), Ministry of Health, Republic of Indonesia. Laporan Nasional RISKESDAS, 2018). This study is registered in the National Institute for Health Research and Development (NIHRD), Ministry of Health, Republic of Indonesia (Kementerian Kesehatan Republik Indonesia, 2021), and permission to access the national surveys data in this study was granted by the NIHRD (SK No. 18052004-119/2020). Ethical approval for the 2013 and 2018 Indonesian Health Surveys was obtained from the Health Research Ethics Committee of NIHRD [(Ref. No. LB.02.01/5.2/KE.006/2013 (2013 survey) and LB.02.01/2/KE.267/2017 (2018 survey)]. Access to the national surveys databases is available upon reasonable request and a thorough review from the NIHRD (National Institute for Health Research and Development (NIHRD), Ministry of Health, Republic of Indonesia, 2021). Written informed consent was obtained from all participants before participating in the survey (National Institute for Health Research and Development (NIHRD), Ministry of Health, Republic of Indonesia. Laporan Nasional RISKESDAS, 2013, National Institute for Health Research and Development (NIHRD), Ministry of Health, Republic of Indonesia. Laporan Nasional RISKESDAS, 2018).

### 2.2 Data collection

An interviewer-assisted questionnaire was used to record information on demographics, lifestyle, and socioeconomic determinants (Kementerian Kesehatan Republik Indonesia, 2013, National Institute for Health Research and Development (NIHRD), Ministry of Health, Republic of Indonesia. Laporan Nasional RISKESDAS, 2013, Kementerian Kesehatan Republik Indonesia, 2018, National Institute for Health Research and Development (NIHRD), Ministry of Health, Republic of Indonesia. Laporan Nasional RISKESDAS, 2018). The questionnaires of the 2013 and 2018 surveys had been published by the Indonesian Ministry of Health previously (Kementerian Kesehatan Republik Indonesia, 2013, National Institute for Health Research and Development (NIHRD), Ministry of Health, Republic of Indonesia. Kuesioner Individu RISKESDAS, 2018). The variables used in this study were measured as described below.

• ***Sociodemography***

Information on sociodemographic characteristics, such as sex, age, urban/rural, education, occupation, and marital status, were obtained from the questionnaire. In this study, we categorised the population into three age groups: young adults (<45 years), middle-aged (45–65 years), and older adults (>65 years) (Geifman et al., 2013 Dec). Urban or rural living situation was determined by the place of residence of the participant as categorised by the Indonesian Central Bureau of Statistics (National Institute for Health Research and Development (NIHRD), Ministry of Health, Republic of Indonesia. Laporan Nasional RISKESDAS, 2013, National Institute for Health Research and Development (NIHRD), Ministry of Health, Republic of Indonesia. Laporan Nasional RISKESDAS, 2018, Dany et al., 2020). The criteria to classify urban or rural areas are population density, availability or accessibility to public facilities (schools, market, hospital, hotel and entertainment centres), and the proportion of households using electricity and telecommunication facilities.

• ***Lifestyle Factors***

Physical activity was reported as frequency (days/week) and duration (minute/day) of moderate and vigorous activity, which we expressed in hours/week. Dietary intake, including fruit and vegetables, was estimated with a simplified semiquantitative food frequency questionnaire as the number of portions eaten per day and then restructured in grams/day (National Institute for Health Research and Development (NIHRD), Ministry of Health, Republic of Indonesia. Kuesioner Individu RISKESDAS, 2018).

Smoking status was assessed as ‘Current’/’Former’/’Never’ categories. Additionally, in the 2018 survey, pack-years of smoking were calculated by multiplying the number of packs of cigarettes smoked per day by the number of years the person smoked (National Institute for Health Research and Development (NIHRD), Ministry of Health, Republic of Indonesia. Kuesioner Individu RISKESDAS, 2018). Alcohol consumption was estimated by the number of portion glasses per day, and then restructured to the unit of millilitre per day. Alcohol consumption was only assessed in the 2018, but not in the 2013 survey. To help participants complete the survey’s questionnaire, display cards of different types of physical activity, cigarettes, as well as typical local dishes and alcohol with different serving sizes were provided by the interviewers as visual aids.

### 2.3 Adherence to the ‘GERMAS’ guideline

Adherence to the ‘GERMAS’ guideline was defined as fulfilling the national healthy lifestyle recommendations, which are (1) physical activity of ≥150 min/week or ≥30 min/day for at least five days, (2) fruit and vegetable consumption of ≥400 g/day (≥5 portions/day), (3) no smoking, and (4) no alcohol consumption (7) (GERMAS, 2021, Kementerian Kesehatan Republik Indonesia, 2017, Kementerian Kesehatan Republik Indonesia, 2016, Kementerian Kesehatan Republik Indonesia, 2016). Only these four out of seven items in the ‘GERMAS’ guideline were investigated in this study, excluding routine health examination, preserving environmental cleanliness, and personal hygiene, as they were more targeted to address the general health state and infectious disease (7). To evaluate the guideline, we dichotomised these exposure variables according to their specific cut-offs and analysed each lifestyle factor separately in relation to the metabolic syndrome. Additionally, we calculated the adherence score as the number of lifestyle recommendations the individuals adhered to and investigated the association of this score with the metabolic syndrome.

### 2.4 Anthropometry

Body weight was measured using a calibrated digital FESCO™ weight scale to the nearest 0.1 kg. Height was measured using a calibrated, vertically fixed tape to the nearest 0.1 cm. BMI was calculated by dividing body weight (kg) by square of height (m^2^). Waist circumference was measured halfway between the iliac crest and lowest rib, using a flexible steel tape to the nearest 0.1 cm (SECA Model 201, Seca Gmbh Co, Hamburg, Germany) (National Institute for Health Research and Development (NIHRD), Ministry of Health, Republic of Indonesia. Laporan Nasional RISKESDAS, 2013, National Institute for Health Research and Development (NIHRD), Ministry of Health, Republic of Indonesia. Laporan Nasional RISKESDAS, 2018). In this study, we categorised the BMI according to the WHO classifications for Asian populations, which are <23.0 kg/m^2^ for normal weight, 23.0–24.9 kg/m^2^ for overweight, and ≥25.0 kg/m^2^ for obesity (World Health Organization, 2000).

### 2.5 Biomarkers

Blood pressure was measured using a digital sphygmomanometer (HEM-7200, Omron Healthcare Co, Ltd, Kyoto, Japan) at the left arm, at an upright sitting position, after 5 min rest. The average of three measurements was used to report participant’s blood pressure. Serum triglyceride and HDL-cholesterol concentrations were determined using standard clinical chemistry methods (in 2013 survey: autoanalyser TRX 7010®, Tokyo Boeki Medical System, LTD. Japan; in 2018 survey: Roche® enzymatic assay) (Dany et al., 2020, National Institute for Health Research and Development (NIHRD), Ministry of Health, Republic of Indonesia. Laporan Nasional RISKESDAS, 2013, National Institute for Health Research and Development (NIHRD), Ministry of Health, Republic of Indonesia. Laporan Nasional RISKESDAS, 2018). Random, fasting, and 2-hour postprandial blood glucose were measured with fingertip capillary blood tests (Accu-Chek Performa, Roche Diagnostics GmbH, Mannheim, Germany). All participants were instructed to fast overnight before blood sampling (National Institute for Health Research and Development (NIHRD), Ministry of Health, Republic of Indonesia. Laporan Nasional RISKESDAS, 2013, National Institute for Health Research and Development (NIHRD), Ministry of Health, Republic of Indonesia. Laporan Nasional RISKESDAS, 2018).

### 2.6 Definition of metabolic syndrome

The metabolic syndrome was defined by the Joint Interim Statement criteria to account for the ethnic-specific cut-off for abdominal obesity in the Asian population (Alberti et al., 2009). Participants were considered to have metabolic syndrome if they had co-occurrence of at least three of the following five cardio-metabolic abnormalities: *abdominal obesity, hypertension, hyperglycemia, hypertriglyceridemia,* and *low HDL-cholesterol* (1)*.* Detailed definitions of the components are shown in **[**Table 1**]**.

**Table 1:** Differences in sociodemographic characteristics, lifestyle factors, and prevalence of metabolic syndrome between the 2013 (n = 34,274) and 2018 (n = 33,786) Indonesian National Health Surveys.

	2013 n = 34,274; 44% men	2018 n = 33,786; 50% men	Difference (95% CI)^3^
Age (Years)	40.1 (15.5)	43.5 (15.8)	3.4 (3.0–3.7)
BMI (kg/m^2^)	22.8 (4.2)	23.7 (4.7)	0.9 (0.8–1.0)
Urban/Rural (%Urban)	50 (49–51)	55 (53–58)	5 (3–8)

**Lifestyle Factors**			
Physically Active (%)	88 (87–89)	79 (78–80)	−9 (-19,-1)
Duration (Hours/Week)^	21.0 (8.3–36.0)	15.0 (3.5–35.0)	−2.5 (-3.3,-1.7)
Adequate Fruit and Vegetable Intake (%)	2 (2–3)	4 (3–4)	2 (1–2)
Fruit and Vegetable Intake (portion/day)^	1.4 (1.0–2.3)	1.4 (0.9–2.3)	0.1 (-0.1,0.1)
Smoking (% Current Smoker)	32 (32–33)	34 (34–35)	2 (1–3)
Alcohol (% Current Drinker)^1^	–	2 (2–2)	

**Metabolic Syndrome Prevalence (%)**^2^	31 (30–32)	32 (31–33)	1 (1–3)
Abdominal Obesity (%)	35 (34–36)	38 (37–39)	3 (1–4)
Hypertension (%)	48 (47–49)	58 (57–59)	10 (9–11)
Hyperglycemia (%)	44 (43–45)	34 (33–35)	−10 (-12,-9)
Hypertriglyceridemia (%)	21 (21–22)	24 (23–25)	3 (2–3)
Low HDL-Cholesterol (%)	41 (40–42)	40 (39–40)	−1 (-3,-1)

Data were reported as mean (SD), median (25th-75th percentiles), or % (95% CI). ^not normally distributed.

Healthy lifestyle was defined by ‘GERMAS’ guideline as ≥ 150 min/week physical activity, ≥5 portions/day fruit and vegetable, no smoking, and no alcohol consumption. Metabolic Syndrome was defined according to the Joint Interim Statement Criteria as the co-occurrence of at least three out of five abnormalities*: (1) abdominal obesity;* waist circumference ≥90 cm for men and ≥80 cm for women, *(2) hypertension*; systolic BP ≥130 mmHg OR diastolic BP ≥80 mmHg, *(3) hyperglycaemia*; fasting glucose ≥140 mg/dL, *(4) hypertriglyceridemia*; triglyceride ≥200 mg/dL, *(5) low HDL-cholesterol*; ≤40 mg/dL in men OR ≤ 50 mg/dL in women.

1 Alcohol consumption was not assessed in the 2013 survey.

2 Analyses were conducted in a subpopulation that was randomly selected for blood glucose measurement; (n = 26,160 in 2013 survey; n = 24,451 in 2018 survey).

3 The standard error of the difference were calculated as sqrt(SE1**2 + SE2**2). The 95% CIs are the estimate +- 1.96 SE.

### 2.7 Statistical analysis

We standardised all estimates for the specific sampling design to represent the general Indonesian population. All analyses were weighted to correct for differences in urban/rural distribution and geographical density across 34 provinces (National Institute for Health Research and Development (NIHRD), Ministry of Health, Republic of Indonesia. Laporan Nasional RISKESDAS, 2013, National Institute for Health Research and Development (NIHRD), Ministry of Health, Republic of Indonesia. Laporan Nasional RISKESDAS, 2018). As a result, percentages are given instead of numbers of participants. For data reporting in this study, we opted for a confidence level of 95% and an alpha of 0.05.

For our first objective, we used the data from the 2013 and 2018 surveys. The lifestyle factors, prevalence of metabolic syndrome, and sociodemographic characteristics were presented as proportions (95% Confidence interval, 95% CI), mean (Standard deviation, SD), or median (25th, 75th percentile). For comparisons, we calculated the differences with 95% confidence intervals between the variables in 2013 and 2018.

For our second objective, we used the data from the subsample of participants who were randomly selected for both blood lipid and glucose tests in the 2018 survey (n = 24,451). We examined the associations of adherence to each lifestyle recommendation in the ‘GERMAS’ guideline with the metabolic syndrome as a binary outcome (metabolic syndrome/no metabolic syndrome), using multivariable logistic regressions categorised by *sex*, *age*, *urban/rural*, and *BMI*, with a different reference category for each categorisation. In detail, the categorised logistic regression analyses that we did were as follows: First, in analysis categorised by sex, we grouped the population into: (1) men who did not adhere to the guideline (reference), (2) men who adhered, (3) women who did not adhere, and (4) women who adhered. We then calculated the prevalence odds ratios (OR) and 95% CI of metabolic syndrome compared to the reference category (non-adherent men), adjusting for age, urban/rural living situation, education, occupation, and marital status. Second, in categorisation by age groups, the population was grouped into: (1) young adults (<45 years) who did not adhere to the guideline (reference), (2) young adults who adhered, (3) middle-aged (45–65 years) who did not adhere, (4) middle-aged who adhered, (5) older adults (>65 years) who did not adhere, and (6) older adults who adhered. The ORs (95% CI) were then compared to the non-adherent young adults as the reference category. Third, in categorisation by urban/rural, we created four groups of (1) non-adherent urban, (2) adherent urban, (3) non-adherent rural, and (4) adherent rural population, and used non-adherent urban as the reference category. Fourth, in categorisation by BMI, we created six groups of (1) non-adherent normal weight (BMI < 23.0 kg/m^2^), (2) adherent normal weight, (3) non-adherent overweight (BMI 23.0–24.9 kg/m^2^), (4) adherent overweight, (5) non-adherent obesity (BMI > 25.0 kg/m^2^), and (6) adherent obesity. We used the non-adherent normal weight group as the reference category.

All associations were adjusted for sociodemographic confounding factors (age as a continuous variable, sex, urban/rural living situation, education, occupation, and marital status). Each lifestyle factor was analysed separately in the regression model, resulting in four estimated odds ratios of metabolic syndrome associated with the separate lifestyle factors. Additionally, in the uncategorised total population, we repeated the regressions with the continuous measures of the lifestyle factors as exposures. We also repeated the regressions with the five separate components of metabolic syndrome as the outcomes.

Finally, we performed three additional analyses. First, as occupational physical activities may have less health benefit than leisure-time physical activity (Holtermann et al., 2018, Dalene et al., 2021), we investigated the duration of physical activity after stratifying by occupation. Second, to examine whether consumption of other foods influenced the association of fruit and vegetable intake with metabolic syndrome, we additionally adjusted the association for unhealthy foods, such as deep-fried foods and sweetened beverages. Third, to investigate whether adherence to multiple recommendations of the guideline was associated with a gradual decrease in risk of metabolic syndrome, we also repeated the regressions with the number of lifestyle recommendations the individuals adhered to as the exposure (‘0’ as adherence to none of the recommendations, to ‘4’ as adherence to all four recommendations). All analyses were performed using STATA (version 16.0, StataCorp, College Station, TX, USA).

## 3 Results

### 3.1 Differences in lifestyle behaviour and metabolic syndrome between the 2013 and 2018 surveys

Lifestyle factors were assessed in participants who were randomly selected for blood lipid tests (n = 34,274 in the 2013 survey; n = 33,786 in the 2018 survey). BMI was higher in the 2018 than in the 2013 population (difference, 95% confidence interval: 0.9, 0.8–1.0 kg/m^2^). The proportion of physically active individuals was lower in the 2018 than in the 2013 population [-9% (-19,-1%)]. In 2013 and 2018, only 2% (2–3%) and 4% (3–4%) of the population consumed an adequate daily amount of fruit and vegetables. The proportion of current smokers were 32% (32–33%) in 2013 and 34% (34–35%) in 2018 **[**Table 1**]**.

Metabolic syndrome and its components were measured in a subsample of participants who were randomly selected for both blood lipid and glucose tests (n = 26,160 in the 2013; n = 24,451 in the 2018 survey). Although the prevalences of metabolic syndrome were similar in 2013 [31% (30–32%)] and 2018 [32% (31–33%)], the contribution of the components was markedly differed. The prevalence of hypertension rose by 10% (9–11%), from 48% (47–49%) to 58% (57–59%), but the prevalence of hyperglycaemia declined by −10% (-12,-9%), from 44% (43–45%) to 34% (33–35%) **[**Table 1**]**.

### 3.2 The associations of adherence to ‘GERMAS’ guideline with metabolic syndrome (in 2018 survey; n = 24,451)

Detailed characteristics of the 2018 survey population, as categorised by sex, age, and urban/rural, were provided in Supplemental Table 1**a and b.** In the uncategorised analysis of the total population, no clear association was observed between adherence to the ‘GERMAS’ guideline and metabolic syndrome **[**Supplemental Table 2**a and b]**. In relation to the components of metabolic syndrome, we observed that adherence to the guideline was associated with lower odds of hypertension [0.88 (0.76–1.02) for fruit and vegetable intake], hyperglycaemia [0.90 (0.83–0.98) for physical activity], hypertriglyceridemia [0.87 (0.79–0.94) for physical activity; 0.83 (0.75–0.92) for no smoking; 0.63 (0.49–0.82) for no alcohol], and low HDL-cholesterol [no smoking: 0.73 (0.67–0.80)]. However, adherence to the guideline was not associated with abdominal obesity **[**Table 2**]**.

**Table 2:** The associations of lifestyle behaviours as recommended in the ‘GERMAS’ guideline with the components of metabolic syndrome (n = 24,451; 2018 Survey).

Lifestyle factors	Proportion (%)	Adjusted odds ratios (95% CI) of the components of metabolic syndrome				
		Abdominal obesity	Hypertension	Hyperglycaemia	Hypertriglyceridemia	Low HDL-Cholesterol
**Physical Activity in hour/week**						
<150 hr/week OR < 30 min/d for 5 days	21	1	1	1	1	1
>150 hr/week OR > 30 min/d for 5 days*	79	1.11 (1.01–1.20)	0.99 (0.91–1.07)	0.90 (0.83–0.98)	0.87 (0.79–0.94)	0.94 (0.87–1.02)

**Fruit & Vegetable Intake**						
<400 g/d (5 portions)	96	1	1	1	1	1
>400 g/d (5 portions)*	4	1.24 (1.05–1.46)	0.88 (0.76–1.02)	1.15 (0.99–1.34)	1.10 (0.94–1.29)	1.07 (0.93–1.23)

**Smoking**						
Current Smoker	34	1	1	1	1	1
Non-Smoker*	66	1.65 (1.48–1.84)	1.46 (1.33–1.60)	0.98 (0.89–1.08)	0.83 (0.75–0.92)	0.73 (0.67–0.80)

**Alcohol Consumption**						
Current Drinker	2	1	1	1	1	1
Non-Drinker*	98	0.98 (0.72–1.34)	0.95 (0.73–1.22)	0.97 (0.74–1.27)	0.63 (0.49–0.82)	1.02 (0.79–1.31)

*As recommended in the healthy lifestyle guideline (‘*GERMAS’*). Data were presented as prevalence odds ratios (OR) with 95% confidence intervals (CI) from the reference category. **For each lifestyle factor, non-adherence to the guideline was set as the reference.** The associations were adjusted for age, sex, urban/rural living situation, education, occupation, and marital status.

**Fig. 1:**
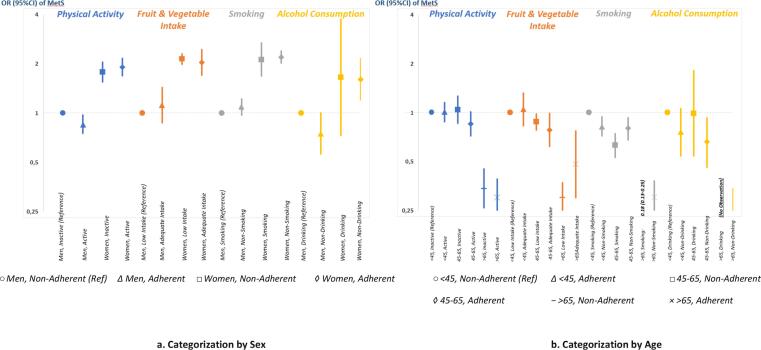

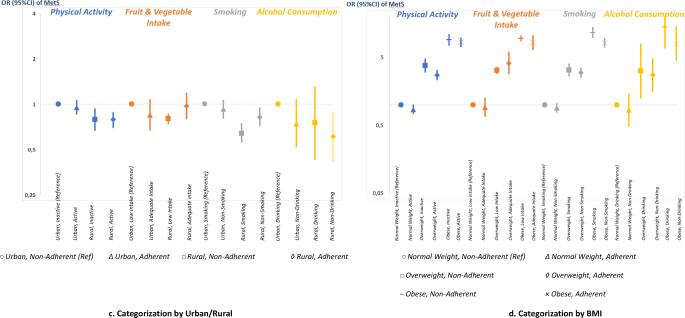
**a, 1b, 1c, 1d.Adherence to the ‘GERMAS’ guideline in relation to the metabolic syndrome; Analyses categorised by Sex (Figure A), by Age (Figure B), by Urban/Rural (Figure C), and by BMI (Figure D) (n = 24,451; 2018 Survey)**. Fig. 1*Data were presented as prevalence OR (95% CI) of the Metabolic Syndrome as compared with the reference category [men; non-adherence (Figure A) age<45; non-adherence (Figure B); urban; non-adherence (Figure C) and normal weight; non-adherence (Figure D)]. Associations were adjusted for age, sex, urban/rural living situation, education, occupation, and marital status. Adherence to the ‘GERMAS’ guideline was defined as fulfilling the national healthy lifestyle recommendations, which are (1) physical activity of >150 minutes/week or >30 minutes/day for at least five days, (2) fruit and vegetable consumption of >400 gram/day (>5 portions), (3) no smoking, and (4) no alcohol consumption*. For BMI-categorization analysis, WHO classifications for Asian populations were used, which were 18.5–22.9 kg/m^2^ for normal weight, 23.0–24.9 kg/m^2^ for overweight, and >25.0 kg/m^2^ for obesity.

When categorising the population by sex, we observed that men who adhered to the guideline had lower odds of metabolic syndrome [adjusted OR (95% CI): 0.85 (0.75–0.97) for physical activity; 0.75 (0.56–1.00) for no alcohol] than men who did not (reference). In the analysis categorised by age, compared with young adults who did not adhere (reference), middle-aged who adhered to the guideline had lower odds of metabolic syndrome [0.85 (0.72–1.01) for physical activity; 0.78 (0.62–0.99) for fruit and vegetable intake; 0.66 (0.46–0.93) for no alcohol]. In categorisation by urban/rural, compared with urban individuals who did not adhere (reference), urban individuals who adhered to the guideline had lower odds of metabolic syndrome [0.85 (0.67–1.07) for fruit and vegetable intake; 0.74 (0.52–1.07) for no alcohol]. In categorisation by BMI, those with overweight or obesity who adhered to the guideline had relatively lower odds of metabolic syndrome than those who did not adhere [e.g., OR (95% CI) of MetS in relation to physical activity: 2.76 (2.35–3.24) in adherent-overweight and 3.80 (3.06–4.73) in nonadherent-overweight; fruit and vegetable intake: 8.25 (6.53–10.43) in adherent-obesity and 9.52 (8.76–10.35) in nonadherent-obesity; no smoking: 8.24 (7.13–9.52) in adherent-obesity and 11.64 (9.82–13.79) in nonadherent-obesity; no alcohol: 2.78 (1.60–4.80) in adherent-overweight and 3.18 (1.28–7.85) in non-adherent overweight. In the categorised analyses, we also observed that women, urban population, and individuals who had overweight or obesity all had higher prevalence odds ratios of metabolic syndrome than the specific reference category for each categorisation **[**Fig. 1**a, b, c, d]**.

### 3.3 Additional analyses

When considering occupation, those with labour-strenuous professions (e.g., farmer, fishers, labour, domestic helper) had a higher duration of physical activity than those without (e.g., student, office workers) **[**Supplemental Table 3**]**. After additionally adjusting for consumption of unhealthy foods, the association of fruit and vegetable intake with metabolic syndrome remained the same [adjusted OR (95% CI): 1.01 (0.87–1.18)]. Consumption of high-risk foods itself was not associated with metabolic syndrome [adjusted OR (95% CI): 0.98 (0.97–1.00)] **[**Supplemental Table 4**].**

Adherence to at least one lifestyle recommendation appeared to be associated with lower odds of metabolic syndrome, and this was explained by the lower odds in three out of five components of metabolic syndrome (hypertension, hypertriglyceridemia, and low HDL-cholesterol). However, we did not find evidence that adherence to two or more lifestyle recommendations was associated with a gradual decrease in the odds of metabolic syndrome [Supplemental Table 5**a, b, c**].

## 4 Discussion

In this study, we aimed to investigate the differences in lifestyle behaviour and prevalence of metabolic syndrome in the Indonesian population between 2013 and 2018, as nationwide health campaigns were performed to introduce the ‘GERMAS’ guideline during 2016–2018. We also investigated the associations between adherence to the guideline and the metabolic syndrome, and how these associations differ between sex, age groups, urban/rural, and BMI categories.

The majority of Indonesian adults were physically active, which appeared mainly due to occupational activities. In the population, fruit and vegetable intake was low. Smoking was common, particularly in men, but alcohol drinking was rare. Despite nationwide health campaigns of the ‘GERMAS’ guideline, no meaningful changes were observed in lifestyle behaviour and prevalence of metabolic syndrome between 2013 and 2018. In our categorised analyses, we observed that women, urban population, and those who had overweight or obesity had increased prevalence odds ratios of metabolic syndrome. In men, the middle-aged, those who had overweight or obesity, and potentially the urban population, adherence to the healthy lifestyle guideline may confer cardiometabolic health benefits.

Our observations were consistent with previous studies from Indonesia, which reported that fruit and vegetable intake was low (2–23%) (Pengpid and Peltzer, 2019, Rachmi et al., 2021, Hermina, 2014), whereas the prevalence of smoking, particularly in men, was high (32–40%) in the population (Holipah et al., 2020, Sujarwoto, 2020). Other studies have also shown that a high proportion of Indonesian adults were physically active (67–74%) (National Institute for Health Research and Development (NIHRD), Ministry of Health, Republic of Indonesia. Laporan Nasional RISKESDAS, 2013, National Institute for Health Research and Development (NIHRD), Ministry of Health, Republic of Indonesia. Laporan Nasional RISKESDAS, 2018), which was similar to our study, although the proportions were lower in children and adolescents (12–52%) (Pengpid and Peltzer, 2019, Andriyani et al., 2020). However, whereas we observed that alcohol consumption was low (2%), another online survey study in 4,584 participants during COVID-19 lockdown in 2020 reported that the prevalence was higher (9.5%), although it was unclear whether the higher consumption of alcohol was associated with the social restriction (Hanafi et al., 2021).

In comparison with studies from other countries, although several studies reported a higher prevalence of obesity in rural populations (Lindroth et al., 2014, Lee et al., 2018), our observations were consistent with most studies in Asian populations, which showed that metabolic syndrome and related diseases were more prevalent in urban than rural areas, partly due to lower physical activity (Park et al., 2004, Song et al., 2015, Weng et al., 2007, Abdul-Rahim et al., 2001, Arambepola et al., 2008, Han et al., 2018). Our results on sex-disparities in metabolic syndrome were also aligned with studies from several populations, which observed that women were more likely to have obesity and metabolic syndrome than men (Park et al., 2004, Wang et al., 2020, Gouveia et al., 2021, Yi and An, 2020, Aekplakorn et al., 2011, Dallongeville et al., 2005). Other cross-sectional and cohort studies investigating similar combined lifestyle factors (physical activity, healthy diet, no smoking, and no alcohol) also observed that adherence to the lifestyle guidelines lowered the risk of metabolic syndrome in men and individuals aged >55 (Garralda-Del-Villar et al., 2018, VanWormer et al., 2017), but not in women and young adults (Garralda-Del-Villar et al., 2018, Bhanushali et al., 2013). This may imply that whereas maintenance of a healthy lifestyle confers a substantial health benefit for men and the middle-aged, more intervention is needed to lower the risk of metabolic syndrome in women and young adults besides a healthy lifestyle.

There are several potential public health implications of our study. Our results suggest that group-specific targeted interventions are needed in Indonesia to prevent metabolic syndrome effectively. For example, interventions to achieve healthy body weight, as well as early screening and prevention programs of metabolic syndrome, may result in a greater gain when targeted to women, the middle-aged, and urban population, as they possess the greatest risk of developing metabolic syndrome. Smoking cessation programs should be aimed at men of all ages and rural populations, as they constitute the majority of smokers in the population. Increasing dietary fruit and vegetable intake in the whole Indonesian population is crucial, and the improvement may particularly lower the odds of metabolic syndrome in the middle-aged and potentially the urban population, as shown in our categorised analyses. Health campaigns to raise awareness of the health benefits of fruit and vegetable intake should be performed. In addition, efforts to reduce domestic prices of fruit and vegetable are pivotal, as unaffordability is reported as a fundamental barrier to adequate fruit and vegetable intake in particularly low-income households in Indonesia (Booth et al., 2019). Overall, the national efforts to promote the ‘GERMAS’ guideline should be maintained, as adhering to the guideline was associated with a cardiometabolic health benefit, particularly in men, the middle-aged, individuals with overweight and obesity, and potentially the urban population. Furthermore, studies have shown that sustaining a healthy lifestyle does not only benefit in preventing the metabolic syndrome, but also slow down its progression to type 2 diabetes and cardiovascular diseases in individuals who already had the metabolic syndrome (Grundy, 2006, Grundy, 2007, Magkos et al., 2009). As adopting a healthy lifestyle may take a longer time to result in health benefits, we recommend that future evaluations of the guideline be done after a longer period. Additionally, as the 2018 survey was the first nationwide survey conducted after the ‘GERMAS’ guideline was released in 2015, we propose that the 2018 survey could be used as the starting point for guideline evaluation in future studies.

The strength of this study is the large and nationally representative study population, which enabled us to generalise our results to the broader Indonesian population. However, several limitations should be mentioned. First, although there were likely a group of participants who were sampled twice in both the 2013 and 2018 surveys, these individuals were not identified, thus no longitudinal data were available. This hindered us from investigating individual changes in the lifestyle behaviour and metabolic syndrome. Second, although the ‘GERMAS’ guideline was promoted widely in national media, it remained unknown whether each participant in this study was exposed to the campaign, thus it is challenging to infer to what extent the national campaign directly influenced the lifestyle behaviour and metabolic syndrome on an individual level. Third, due to the nature of data collection, information bias or possible measurement error could not be excluded as participants may provide socially desirable answers for the lifestyle questionnaires. Fourth, several important variables were not measured in the surveys, such as alcohol consumption and pack-years of smoking in the 2013 survey, and the use of lipid-lowering medications in both surveys. Whereas we have incorporated the use of anti-hypertensive and glucose-lowering medications in the components hypertension and hyperglycaemia, the unavailability of information on lipid-lowering medications may result in an underestimation of the prevalence of hypertriglyceridemia and low HDL-cholesterol in this study. Nevertheless, as lipid-lowering medications are not routinely prescribed in Indonesia (Pramono and Harbuwono, 2015), we expect that this possible underestimation would not have influenced our results dramatically. Fifth, our results only pertain to the relationship between adherence to the ‘GERMAS’ guideline and metabolic syndrome, hence, whether adherence to the guideline may offer a greater benefit in preventing other diseases remains to be investigated. Sixth, as Indonesians generally have a relatively low life expectancy (72 years) (The World Bank, 2022), this may imply that older adults who were sampled in the study were ‘healthy survivors’, or may have had healthier lifestyle behaviour in the past. This may potentially explain the low prevalence odds ratios of metabolic syndrome in older adults as observed in our study. Seventh, as we observed no clear association between adherence to the guideline and metabolic syndrome in the total population, but some associations in several subgroups, this may imply that confounding by age and sex possibly exist. However, we performed the analyses categorised by sex and age, so the potential effect of these confounders can be controlled. Due to the observational nature of our study, we cannot completely exclude either that the results may have been influenced by residual confounding factors, which may distort the true exposure-outcome associations (Egger et al., 1998). Lastly, we acknowledge that testing multiple hypotheses may increase the risk of false-positive findings. Nevertheless, according to our a priori hypothesis, as we investigated four associations in the total population as categorised by sex, age groups, urban/rural, and BMI categories, the corresponding probability of type I error in at least one test is relatively minimal (Groenwold et al., 2021). Taken together, despite the mentioned limitations, the national health surveys were the largest and the best currently available health database to represent the heterogenous Indonesian population, thus these limitations should not outweigh the importance to report the study observations.

In conclusion, we observed that lifestyle behaviour and the prevalence of metabolic syndrome in Indonesia did not markedly change between 2013 and 2018. Adherence to the ‘GERMAS’ guideline may benefit the cardiometabolic health of men, the middle-aged, those with overweight and obesity, and potentially the urban population. Nationwide health campaign of ‘GERMAS’ should be maintained, and more emphasis on improving the low fruit and vegetable intake is crucial. Our observations also suggest that sociodemographic differences should be taken into account when designing public health strategies to effectively prevent metabolic syndrome in the population. As Indonesia is a multi-ethnic nation that may have different lifestyle behaviours, types of staple foods and fruit and vegetable preferences per region, local approaches to promote the guideline may be beneficial.

### CRediT authorship contribution statement

**Fathimah S. Sigit:** Conceptualization, Formal analysis, Methodology, Visualization, Writing – original draft. **Stella Trompet:** Conceptualization, Methodology, Supervision, Writing – review & editing. **Dicky L. Tahapary:** Conceptualization, Methodology, Writing – review & editing. **Dante S. Harbuwono:** Conceptualization, Methodology, Writing – review & editing. **Saskia le Cessie:** Conceptualization, Methodology, Writing – review & editing. **Frits R. Rosendaal:** Conceptualization, Methodology, Supervision, Writing – review & editing. **Renée de Mutsert:** Conceptualization, Methodology, Supervision, Writing – review & editing.

## Declaration of Competing Interest

The authors declare that they have no known competing financial interests or personal relationships that could have appeared to influence the work reported in this paper.
