# Supplementary material for: Adherence to the healthy lifestyle guideline in relation to the metabolic syndrome: Analyses from the 2013 and 2018 Indonesian national health surveys
Source: Prev Med Rep. 2022 Apr 27;27:101806. doi: 10.1016/j.pmedr.2022.101806 (PMC9152785; doi:10.1016/j.pmedr.2022.101806)
Supplement: Supplementary data 1 [file mmc1.docx]

**Supplementary Tables and Figure**

**Supplemental Table 1a.** Descriptive characteristics of the Indonesian population, as categorized by Age and Sex (n= 33,786; 2018 Survey)

**Supplemental Table 1b.** Descriptive characteristics of the Indonesian population, as categorized by Urban/Rural (n= 33,786; 2018 Survey)

**Supplemental Table 2a.** The associations of lifestyle behaviour as recommended in the ‘GERMAS’ guideline with the metabolic syndrome; Uncategorized analysis in the total population (n=24,451; 2018 Survey)

**Supplemental Table 2b.** The associations of the continuous measures of lifestyle behaviour as recommended in the ‘GERMAS’ guideline with the metabolic syndrome; Uncategorized analysis in the total population (n=24,451; 2018 Survey)

**Supplemental Table 3.** Physical Activity in the Population, as Stratified by Occupations (2013 and 2018 Survey)

**Supplemental Table 4.** Consumption of High-Risk Foods (n=33,786; 2018 Survey), and the Associations of Fruit & Vegetable Intake with the Metabolic Syndrome after Additional Adjustment for High-Risk Foods (n=24,451; 2018 Survey)

**Supplemental Table 5a.** Adherence score in relation to the metabolic syndrome (n=24,451; 2018 Survey)

**Supplemental Table 5b.** Adherence score in relation to the components of metabolic syndrome (n=24,451; 2018 Survey)

**Supplemental Table 5c.** Adherence score in relation to the metabolic syndrome. Analyses categorized by sex, by age, by urban/rural, and by BMI (n=24,451; 2018 Survey)

**Supplemental Figure 1.** Study Flow Chart

**Supplemental Table 1a. Descriptive characteristics of the Indonesian population, as categorized by Age and Sex (n= 33,786; 2018 Survey)**

|  | **Age <45 (53%)** | | **Age 45-65 (39%)** | | **Age >65 (8%)** | |
| --- | --- | --- | --- | --- | --- | --- |
|  | Men (48%) | Women (52%) | Men (53%) | Women (47%) | Men (56%) | Women(44%) |
| **Sociodemographic** |  |  |  |  |  |  |
| Age (Years) | 30.9 (8.2) | 31.5 (9.2) | 54.1 (5.3) | 53.3 (6.2) | 72.5 (5.3) | 72.4 (6.5) |
| BMI (kg/m^2^)^1^ | 22.5 (3.9) | 24.9 (5.4) | 23.1 (3.7) | 25.3 (5.3) | 21.0 (3.3) | 22.5 (4.9) |
| Education (% High) | 6 (6-7) | 6 (6-7) | 6 (6-7) | 4 (4-5) | 5 (4-7) | 2 (1-3) |
| Marital Status (% Married) | 64 (62-65) | 79 (78-80) | 92 (92-93) | 78 (77-79) | 80 (78-82) | 34 (32-37) |
| Urban/Rural (% Urban) | 57 (55-60) | 57 (54-59) | 54 (51-57) | 55 (52-57) | 47 (44-51) | 49 (45-52) |
| **Lifestyle Factors** |  |  |  |  |  |  |
| Physically Active (%) | 76 (74-77) | 83 (82-84) | 80 (78-81) | 83 (82-85) | 66 (63-68) | 60 (57-63) |
| Duration (hr/wk)^ | 17.5 (2.5-42.0) | 14.0 (5.3-28.0) | 21.0 (4.0-42.0) | 15.0 (6.0-30.3) | 9.0 (0.0-28.0) | 6.0 (0.0-18.0) |
| Adequate Fruit-Veg Intake (%) | 3 (3-4) | 3 (3-4) | 4 (4-5) | 4 (4-5) | 4 (3-5) | 4 (3-5) |
| Qty (portion/day)^ | 1.3 (0.9-2.1) | 1.4 (1.0-2.3) | 1.4 (1.0-2.4) | 1.4 (1.0-2.5) | 1.3 (0.9-2.1) | 1.4 (0.9-2.3) |
| Smoking (% Current) | 67 (66-68) | 2 (2-3) | 65 (63-66) | 4 (3-5) | 55 (52-58) | 5 (4-6) |
| Pack years^^2^ | 6.6 (2.6-12.0) | 2.4 (0.7-6.0) | 18.8 (10.8-26.4) | 5.7 (2.7-12.3) | 24.0 (12.5-33.6) | 7.5 (3.9-16.5) |
| Alcohol (% Current) | 6 (5-7) | 1 (0-1) | 2 (1-2) | 1 (0-1) | 1 (0-1) | 0 (0-0) |
| **Comorbidities** |  |  |  |  |  |  |
| History of Cardiovascular Diseases (%) | 1 (1-1) | 1 (1-2) | 3 (3-4) | 4 (3-4) | 4 (3-6) | 5 (4-7) |
| History of Stroke (%) | 1 (0-1) | 1 (0-1) | 2 (2-3) | 2 (2-2) | 3 (2-4) | 3 (2-4) |
| History Diabetes (%) | 1 (1-2) | 2 (1-2) | 6 (6-7) | 8 (7-8) | 6 (5-8) | 8 (7-10) |
| Use of anti-hypertensive medication(s) (%) | 2 (2-3) | 5 (4-5) | 10 (9-11) | 21 (20-22) | 17 (15-19) | 28 (25-30) |
| Use of oral diabetic medication(s) or insulin (%) | 1 (1-1) | 1 (1-1) | 5 (4-6) | 6 (6-7) | 4 (3-6) | 7 (6-8) |
| **Metabolic Syndrome (%)^3^** | 19 (18-21) | 30 (29-31) | 30 (28-32) | 50 (48-51) | 24 (21-27) | 46 (43-50) |
| Abdominal Obesity^4^ | 15 (14-17) | 49 (48-51) | 23 (22-25) | 61 (60-63) | 15 (12-17) | 46 (43-49) |
| Hypertension | 41 (39-43) | 47 (45-48) | 65 (64-67) | 75 (74-76) | 75 (72-78) | 86 (84-88) |
| Hyperglycemia | 25 (24-27) | 23 (22-24) | 41 (39-43) | 46 (45-48) | 47 (44-51) | 52 (48-55) |
| Hypertriglyceridemia | 26 (24-27) | 14 (14-15) | 32 (30-33) | 28 (26-29) | 22 (20-25) | 28 (25-31) |
| Low HDL-Cholesterol | 33 (31-35) | 51 (50-53) | 29 (28-31) | 43 (42-45) | 23 (20-26) | 39 (36-42) |
| Waist Circumference (cm)^4^ | 77.7 (10.7) | 79.7 (12.7) | 81.2 (10.8) | 83.1 (13.6) | 77.2 (10.9) | 78.7 (15.4) |
| Systolic Blood Pressure (mmHg) | 123.6 (13.6) | 121.8 (19.9) | 137.7 (21.7) | 143.2 (28.9) | 147.0 (25.0) | 157.2 (33.1) |
| Diastolic Blood Pressure (mmHg) | 81.1 (9.8) | 83.4 (13.0) | 86.1 (12.1) | 88.7 (14.8) | 83.5 (12.6) | 86.1 (16.6) |
| Fasting Plasma Glucose (mmol/L) | 5.3 (0.9) | 5.4 (1.4) | 5.8 (1.6) | 6.1 (2.6) | 5.9 (1.6) | 6.1 (2.3) |
| Serum Triglyceride (mmol/L) | 1.6 (1.0) | 1.2 (0.8) | 1.7 (1.2) | 1.6 (1.1) | 1.4 (0.8) | 1.6 (1.0) |
| HDL-Cholesterol (mmol/L) | 1.1 (0.2) | 1.3 (0.3) | 1.2 (0.2) | 1.3 (0.3) | 1.2 (0.3) | 1.4 (0.4) |

Data were presented as mean (SD), median (25^th^-75^th^ percentiles), or % (95% CI). ^not normally distributed. Results were based on analyses weighted towards geographical density across 34 provinces in Indonesia.

^1^WHO BMI cut-offs were >23 kg/m^2^ for overweight and >25 kg/m^2^ for obesity in the Asian population.

^2^Median (25^th^-75^th^ percentiles) was calculated from individuals who smoke

^3^Metabolic Syndrome was defined by the Joint Interim Statement Criteria as the co-occurrence of at least three out of five abnormalities*: (1) abdominal obesity*, *(2) hypertension*; systolic BP >130 mmHg OR diastolic BP >80 mmHg, *(3) hyperglycaemia*; fasting glucose >140 mg/dL, *(4) hypertriglyceridemia*; triglyceride >200 mg/dL, *(5) low HDL-cholesterol*; <40 mg/dL in men OR <50 mg/dL in women. The prevalence was estimated from individuals who were randomly selected for blood glucose measurements (n=24,451)

^4^Ethnic-Specific (Asian) waist-circumference cut-offs for abdominal obesity were >90 cm for men and >80 cm for women.

**Supplemental Table 1b.** **Descriptive characteristics of the Indonesian population, as categorized by Urban/Rural (n= 33,786; 2018 Survey)**

|  | **Urban (55%)** | **Rural (45%)** |
| --- | --- | --- |
| **Sociodemographic** |  |  |
| Age (Years) | 42.7 (14.9) | 44.5 (16.7) |
| BMI (kg/m^2^)^1^ | 24.3 (4.7) | 23.0 (4.6) |
| Education (% High) | 8 (8-9) | 3 (2-3) |
| Marital Status (% Married) | 74 (73-75) | 79 (78-80) |
| **Lifestyle Factors** |  |  |
| Physically Active (%) | 73 (72-75) | 85 (84-86) |
| Duration (hour/week)^ | 11.3 (2.0-28.0) | 21.0 (7.0-42.0) |
| Adequate Fruit & Veg Intake (%) | 3 (3-4) | 4 (4-5) |
| Qty (portion/day)^ | 1.4 (0.9-2.1) | 1.4 (1.0-2.6) |
| Smoking (% Current) | 32 (31-33) | 37 (36-38) |
| Pack years^^2^ | 10.2 (4.2-19.2) | 12.0 (5.8-21.5) |
| Alcohol (% Current) | 2 (2-3) | 2 (2-2) |
| **Comorbidities** |  |  |
| History of Cardiovascular Diseases (%) | 3 (2-3) | 2 (2-2) |
| History of Stroke (%) | 1 (1-2) | 1 (1-1) |
| History Diabetes (%) | 5 (5-6) | 3 (3-3) |
| Use of anti-hypertensive medication(s) (%) | 11 (10-11) | 8 (8-9) |
| Use of oral diabetic medication(s) or insulin (%) | 4 (4-4) | 2 (2-2) |
| **Metabolic Syndrome (%)^3^** | 34 (33-35) | 30 (29-31) |
| Abdominal Obesity^4^ | 44 (43-46) | 30 (29-31) |
| Hypertension | 58 (57-59) | 58 (57-59) |
| Hyperglycemia | 34 (33-36) | 34 (33-35) |
| Hypertriglyceridemia | 24 (24-25) | 23 (22-24) |
| Low HDL-Cholesterol | 39 (38-40) | 40 (39-41) |
| Waist Circumference (cm)^4^ | 81.8 (11.8) | 77.7 (12.2) |
| Systolic Blood Pressure (mmHg) | 131.4 (23.0) | 132.8 (25.1) |
| Diastolic Blood Pressure (mmHg) | 84.8 (12.5) | 84.1 (13.2) |
| Fasting Plasma Glucose (mmol/L) | 5.7 (1.8) | 5.6 (1.6) |
| Serum Triglyceride (mmol/L) | 1.5 (1.1) | 1.4 (1.0) |
| HDL-Cholesterol (mmol/L) | 1.2 (0.3) | 1.2 (0.3) |

Data were presented as mean (SD), median (25^th^-75^th^ percentiles), or percentage. ^not normally distributed. Results were based on analyses weighted towards geographical density across 34 provinces in Indonesia.

^1^WHO BMI cut-offs were >23 kg/m^2^ for overweight and >25 kg/m^2^ for obesity in the Asian population.

^2^Median (25^th^-75^th^ percentiles) was calculated from individuals who smoke

^3^Metabolic Syndrome was defined by the Joint Interim Statement Criteria as the co-occurrence of at least three out of five abnormalities*: (1) abdominal obesity*, *(2) hypertension*; systolic BP >130 mmHg OR diastolic BP >80 mmHg, *(3) hyperglycaemia*; fasting glucose >140 mg/dL, *(4) hypertriglyceridemia*; triglyceride >200 mg/dL, *(5) low HDL-cholesterol*; <40 mg/dL in men OR <50 mg/dL in women. The prevalence was estimated from individuals who were randomly selected for glucose measurements (n=24,451)

^4^Ethnic-Specific (Asian) waist-circumference cut-offs for abdominal obesity were >90 cm for men and >80 cm for women.

**Supplemental Table 2a. The associations of lifestyle behaviour as recommended in the ‘GERMAS’ guideline with the metabolic syndrome; Uncategorized analysis in the total population (n=24,451; 2018 Survey)**

| **Lifestyle Factors** | **Proportion (%)** | **Prevalence Odds Ratios (95% CI) of Metabolic Syndrome** | |
| --- | --- | --- | --- |
|  |  | Model 1 | Model 2 |
| **Physical Activity** |  |  |  |
| <150 min/week OR <30 min/d for 5 days | 21 | 1 | 1 |
| >150 min/week OR >30 min/d for 5 days* | 79 | 0.91 (0.84-0.99) | 0.97 (0.89-1.05) |
| **Fruit & Vegetable Intake** |  |  |  |
| <400 g/d (5 portions) | 96 | 1 | 1 |
| >400 g/d (5 portions)* | 4 | 1.03 (0.89-1.20) | 1.02 (0.88-1.19) |
| **Smoking** |  |  |  |
| Current Smoker | 34 | 1 | 1 |
| Non-Smoker* | 66 | 1.14 (1.03-1.26) | 1.08 (0.98-1.19) |
| **Alcohol Consumption** |  |  |  |
| Current Drinker | 2 | 1 | 1 |
| Non-Drinker* | 98 | 0.76 (0.57-1.00) | 0.77 (0.58-1.01) |

*As recommended in the healthy lifestyle guideline (*Gerakan Masyarakat Hidup Sehat,* ‘*GERMAS’*). Abbreviations: g/d, gram/day; min/week, minute/week; min/d, minute/day.

Healthy lifestyle was defined by ‘GERMAS’ guideline as >150 minutes/week physical activity, >5 portions/day fruit and vegetable, no smoking, and no alcohol consumption. Data were presented as prevalence odds ratios (OR) with 95% confidence intervals (CI) from the reference category. Each lifestyle factor was analyzed separately in the regression model. **For each lifestyle factor, non-adherence to the guideline was set as the reference.**

Model 1: Adjusted for age and sex

Model 2: Adjusted for Model 1 + other confounders (urban/rural living situation, education, occupation, marital status)

**Supplemental Table 2b. The associations of the continuous measures of lifestyle behaviour as recommended in the ‘GERMAS’ guideline with the metabolic syndrome; Uncategorized analysis in the total population (n=24,451; 2018 Survey)**

| **Lifestyle Factors** | **Prevalence Odds Ratios (95% CI) of Metabolic Syndrome** | | |
| --- | --- | --- | --- |
|  | Crude | Model 1 | Model 2 |
| **Using the actual units** |  |  |  |
| Physical Activity (hour/week) | 0.99 (0.99-1.00) | 1.00 (0.99-1.00) | 1.00 (1.00-1.00) |
| Fruit & Vegetable Intake (portion/day) | 1.04 (1.02-1.06) | 1.03 (1.00-1.05) | 1.02 (1.00-1.05) |
| Smoking (Pack Years) | 1.01 (1.01-1.02) | 1.01 (1.00-1.01) | 1.01 (1.00-1.01) |
| Alcohol Consumption (mL/day) | 1.00 (1.00-1.00) | 1.00 (1.00-1.00) | 1.00 (1.00-1.00) |
|  |  |  |  |
| **Using the standardized (SD) units*** |  |  |  |
| Physical Activity (SD) | 0.89 (0.86-0.92) | 0.92 (0.89-0.95) | 0.95 (0.92-0.98) |
| Fruit & Vegetable Intake (SD) | 1.05 (1.02-1.08) | 1.04 (1.01-1.07) | 1.03 (1.00-1.06) |
| Smoking (SD) | 1.18 (1.10-1.26) | 1.12 (1.03-1.21) | 1.12 (1.03-1.21) |
| Alcohol Consumption (SD) | 1.01 (0.97-1.04) | 1.03 (1.00-1.06) | 1.03 (1.00-1.06) |

Data were presented as prevalence odds ratios (OR) with 95% confidence intervals (CI). Each lifestyle factor was analyzed separately in the regression model.

Model 1: Adjusted for age and sex

Model 2: Adjusted for Model 1 + other confounders (urban/rural living situation, education, occupation, marital status)

*1 SD: 21.1 hours/week physical activity; 1.4 portion/day fruit and vegetable; 15.1 pack-years of smoking; and 70.8 mL/day alcohol.

**Supplemental Table 3. Physical Activity in the Population, as Stratified by Occupations (2013 and 2018 Survey)**

| **Occupation** | **Proportion from Total Population (%)** | **Physically Active (%)** | **Duration of Physical Activity** |
| --- | --- | --- | --- |
|  |  |  |  |
| **2018 Survey; n=33,786** |  |  |  |
| Unemployed/Retired/Stay-at-Home | 32 | 75 | 11.0 (2.3-21.0) |
| Currently in Education | 5 | 58 | 3.8 (0.5-14.0) |
| Civil Servant/Army/Police | 2 | 70 | 10.0 (1.3-28.0) |
| Private Employees | 8 | 72 | 12.0 (1.8-37.5) |
| Entrepreneur | 15 | 79 | 15.8 (3.5-35.0) |
| Farmer | 21 | 91 | 28.0 (14.0-44.3) |
| Fishermen | 1 | 94 | 36.2 (24.0-60.7) |
| Labor/Driver/Domestic Helper | 12 | 84 | 24.0 (7.0-45.0) |
| Other | 5 | 75 | 14.0 (2.3-31.5) |
|  |  |  |  |
| **2013 Survey; n=20,345^1^** |  |  |  |
| Civil Servant/Army/Police | 4 | 86 | 21.0 (7.0-35.0) |
| Private Employees | 14 | 86 | 24.0 (7.5-42.0) |
| Entrepreneur | 22 | 91 | 24.5 (11.0-42.0) |
| Farmer | 31 | 94 | 28.0 (14.1-42.0) |
| Fishermen | 1 | 97 | 33.0 (20.0-42.0) |
| Labour | 22 | 92 | 30.0 (14.0-45.0) |
| Other | 6 | 91 | 25.5 (12.5-41.5) |

Data were presented percentage (%) and median (25^th^-75^th^ percentiles). Results were based on analyses weighted towards geographical density across 34 provinces in Indonesia.

^1^There were 13,929 missing observations in the 2013 survey on the type of occupation.

**Supplemental Table 4. Consumption of High-Risk Foods (n=33,789; 2018 Survey), and the Associations of Fruit & Vegetable Intake with the Metabolic Syndrome after Additional Adjustment for High-Risk Foods (n=24,451; 2018 Survey)**

| **Type of Foods** | | **Quantity (portion/day)** | |  |
| --- | --- | --- | --- | --- |
| Sweets/Sugary Foods | | 0.6 (0.2-1.0) | |  |
| Sweetened Beverages | | 1.0 (0.2-2.0) | |  |
| Salty Foods | | 0.6 (0.2-1.0) | |  |
| Deep-Fried/Fatty Foods | | 0.6 (0.2-2.0) | |  |
| Grilled Foods | | 0.1 (0.0-0.2) | |  |
| Meat/Chicken/Fish with Preservatives | | 0.0 (0.0-0.1) | |  |
| Food Additives/MSG | | 1.0 (1.0-2.0) | |  |
| Soft/Carbonated Drinks | | 0.0 (0.0-0.1) | |  |
| Energy Drinks | | 0.0 (0.0-0.0) | |  |
| Instant Noodles/Other Instant Foods | | 0.2 (0.1-0.6) | |  |
|  |  |  |  |  |
|  | **Prevalence Odds Ratios (95% CI) of Metabolic Syndrome** | | | |
|  | **Crude** | **Model 1** | **Model 2** |  |
| **High-Risk Foods** | 0.95 (0.94-0.97) | 0.98 (0.97-1.00) | 0.98 (0.97-1.00) |  |
|  |  |  |  |  |
|  | **Prevalence Odds Ratios (95% CI) of Metabolic Syndrome** | | | |
|  | **Crude** | **Model 1** | **Model 2** | **Model 3** |
| **Fruit & Vegetable Intake** |  |  |  |  |
| <400 g/d (5 portions) | Ref | Ref | Ref | Ref |
| >400 g/d (5 portions)* | 1.06 (0.92-1.23) | 1.03 (0.89-1.20) | 1.02 (0.88-1.19) | 1.01 (0.87-1.18) |

*As recommended in the healthy lifestyle guideline (‘*GERMAS’*)

Data were presented as median (25^th^-75^th^ percentiles) and prevalence odds ratios (OR) with 95% confidence intervals (CI) from the reference category.

Model 1: Adjusted for age and sex

Model 2: Adjusted for Model 1 + other confounders (urban/rural living situation, education, occupation, marital status)

Model 3: Adjusted for Model 2 + **consumption of high-risk foods** (sugar-added foods, sweetened beverages, salty foods, deep-fried foods, grilled foods, meat with preservatives, food additives, carbonated drinks, energy drinks, instant foods)

**Supplemental Table 5a. Adherence score in relation to the metabolic syndrome (n=24,451; 2018 Survey)**

| Adherence score* | Proportion (%) | Prevalence Odds Ratios (95% CI) of Metabolic Syndrome | |
| --- | --- | --- | --- |
|  |  | Model 1 | Model 2 |
| 0 | 1 | 1 | 1 |
| 1 | 6 | 0.72 (0.38-1.36) | 0.72 (0.38-1.38) |
| 2 | 36 | 0.66 (0.36-1.23) | 0.69 (0.37-1.30) |
| 3 | 55 | 0.69 (0.37-1.28) | 0.72 (0.38-1.35) |
| 4 | 2 | 0.66 (0.35-1.26) | 0.68 (0.35-1.31) |

Data were presented as OR (95% CI) from the reference category. The adherence score represents the number of lifestyle behaviour that fulfil the ‘GERMAS’ guideline.

Model 1: Adjusted for age and sex

Model 2: Adjusted for Model 1 + other confounders (urban/rural living situation, education, occupation, marital status)

**Supplemental Table 5b. Adherence score in relation to the components of metabolic syndrome (n=24,451; 2018 Survey)**

| **Adherence score*** | **Proportion (%)** | **Adjusted Odds Ratios (95% CI) of the Components of Metabolic Syndrome** | | | | |
| --- | --- | --- | --- | --- | --- | --- |
|  |  | **Abdominal Obesity** | **Hypertension** | **Hyperglycaemia** | **Hypertriglyceridemia** | **Low HDL-Cholesterol** |
| **0** | 1 | 1 | 1 | 1 | 1 | 1 |
| **1** | 6 | 1.37 (0.66-2.85) | 0.61 (0.35-1.06) | 1.12 (0.60-2.10) | 0.53 (0.30-0.93) | 0.92 (0.52-1.62) |
| **2** | 36 | 1.46 (0.71-2.97) | 0.70 (0.40-1.22) | 1.11 (0.60-2.06) | 0.48 (0.27-0.84) | 0.75 (0.43-1.31) |
| **3** | 55 | 2.01 (0.98-4.12) | 0.82 (0.47-1.44) | 1.02 (0.55-1.89) | 0.40 (0.23-0.71) | 0.66 (0.37-1.15) |
| **4** | 2 | 2.18 (1.05-4.53) | 0.73 (0.41-1.32) | 1.17 (0.62-2.23) | 0.40 (0.22-0.73) | 0.68 (0.38-1.22) |

Data were presented as OR (95% CI) from the reference category. The adherence score represents the number of lifestyle behaviour that fulfil the ‘GERMAS’ guideline. The associations were adjusted for age, sex, urban/rural living situation, education, occupation, and marital status.

**Supplemental Table 5c. Adherence score in relation to the metabolic syndrome. Analyses categorized by sex, by age, by urban/rural, and by BMI (n=24,451; 2018 Survey)**

| **Adherence Score*** | **Adjusted Odds Ratios (95% CI) of Metabolic Syndrome** | | | | | | | | | |
| --- | --- | --- | --- | --- | --- | --- | --- | --- | --- | --- |
|  | **Categorization by Sex** | | | | **Categorization by Age** | | | | | |
|  | **Proportion (%)** | **Men** | **Proportion (%)** | **Women** | **Proportion (%)** | **Young Adults** | **Proportion (%)** | **Middle-Aged** | **Proportion (%)** | **Older Adults** |
| **0** | 1 | 1 | 0 | 1 | 1 | 1 | 1 | 1 | 0 | 1 |
| **1** | 13 | 0.88 (0.44-1.76) | 1 | 0.06 (0.01-1.29) | 6 | 0.71 (0.32-1.58) | 6 | 0.89 (0.23-3.48) | 9 |  |
| **2** | 56 | 0.86 (0.44-1.69) | 19 | 0.11 (0.01-2.12) | 34 | 0.62 (0.29-1.32) | 37 | 0.82 (0.21-3.20) | 46 | 1.10 (0.71-1.73) |
| **3** | 28 | 0.84 (0.43-1.66) | 77 | 0.11 (0.01-2.19) | 57 | 0.58 (0.27-1.25) | 55 | 0.81 (0.21-3.17) | 43 | 1.13 (0.71-1.81) |
| **4** | 2 | 0.76 (0.35-1.68) | 3 | 0.11 (0.01-2.14) | 2 | 0.61 (0.27-1.37) | 3 | 0.63 (0.16-2.51) | 2 | 1.38 (0.68-2.80) |

| **Adherence Score*** | **Adjusted Odds Ratios (95% CI) of Metabolic Syndrome** | | | | | | | | | |
| --- | --- | --- | --- | --- | --- | --- | --- | --- | --- | --- |
|  | **Categorization by Urban/Rural** | | | | **Categorization by BMI** | | | | | |
|  | **Proportion (%)** | **Urban** | **Proportion (%)** | **Rural** | **Proportion (%)** | **Normal Weight** | **Proportion (%)** | **Overweight** | **Proportion (%)** | **Obesity** |
| **0** | 1 | 1 | 1 | 1 | 1 | 1 | 1 | 1 | 1 | 1 |
| **1** | 8 | 0.72 (0.33-1.59) | 5 | 0.75 (0.23-2.44) | 8 | 0.72 (0.22-2.40) | 5 | 0.85 (0.16-4.46) | 4 | 0.26 (0.05-1.31) |
| **2** | 35 | 0.73 (0.34-1.56) | 37 | 0.72 (0.23-2.29) | 44 | 0.69 (0.21-2.24) | 35 | 0.63 (0.13-3.18) | 27 | 0.24 (0.05-1.22) |
| **3** | 54 | 0.73 (0.34-1.56) | 56 | 0.75 (0.23-2.42) | 46 | 0.55 (0.17-1.78) | 57 | 0.48 (0.10-2.44) | 66 | 0.22 (0.04-1.08) |
| **4** | 2 | 0.55 (0.24-1.22) | 3 | 0.86 (0.26-2.83) | 2 | 0.30 (0.08-1.07) | 3 | 0.75 (0.14-4.04) | 3 | 0.18 (0.03-0.90) |

Data were presented as OR (95% CI) from the reference category. The adherence score represents the number of lifestyle behaviour that fulfil the ‘GERMAS’ guideline. The associations were adjusted for age, sex, urban/rural living situation, education, occupation, and marital status.


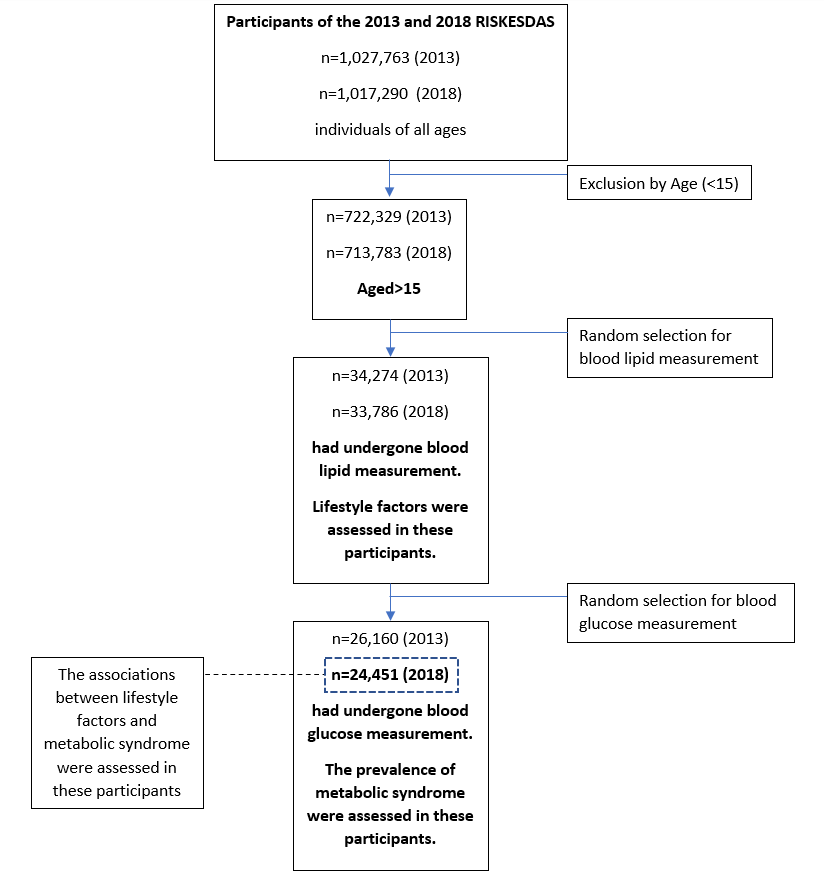


**Supplemental Figure 1. Study Flow Chart**
